# 3-Oxo-*N*′,2-diphenyl-2,3-di­hydro-1*H*-pyrazole-4-carbohydrazide

**DOI:** 10.1107/S1600536814012392

**Published:** 2014-06-25

**Authors:** Joel T. Mague, Shaaban K. Mohamed, Mehmet Akkurt, Eman A. Ahmed, Mustafa R. Albayati

**Affiliations:** a Department of Chemistry, Tulane University, New Orleans, LA 70118, USA; b Chemistry and Environmental Division, Manchester Metropolitan University, Manchester M1 5GD, England; c Chemistry Department, Faculty of Science, Minia University, 61519 El-Minia, Egypt; d Department of Physics, Faculty of Sciences, Erciyes University, 38039 Kayseri, Turkey; e Chemistry Department, Faculty of Science, Sohag University, 61519 Sohag, Egypt; f Kirkuk University, College of Science, Department of Chemistry, Kirkuk, Iraq

## Abstract

In the title compound, C_16_H_14_N_4_O_2_, the pyrazole ring makes a dihedral angle of 10.49 (8)° with its N-bound phenyl group, while it is nearly perpendicular to the other phenyl ring [dihedral angle = 88.47 (5)°]. The mol­ecular conformation is stabilized by intra­molecular C—H⋯O and N—H⋯O hydrogen bonds. In the crystal, the packing involves sheets of mol­ecules parallel to (100) linked by N—H⋯O hydrogen bonds. A C—H⋯O interaction is also observed.

## Related literature   

For the diverse biological activities of pyrazolone compounds, see: Guckian *et al.* (2010 ▶); Fan *et al.* (2006 ▶); Castagnolo *et al.* (2008 ▶); Idrees *et al.* (2009 ▶); Abdel-Aziz *et al.* (2009 ▶); Manojkumar *et al.* (2009 ▶); Shete *et al.* (2011 ▶); Sujatha *et al.* (2009 ▶); El-Hawash *et al.* (2006 ▶); Kawai *et al.* (1997 ▶); Wu *et al.* (2002 ▶6). For industrial applications of pyrazolo­nes, see: Basaif *et al.* (2007 ▶); Ho (2005 ▶); Kirschke *et al.* (1984 ▶); Chande *et al.* (1993 ▶); El-Saraf & El-Sayed (2003 ▶). For graph-set motif notation, see: Bernstein *et al.* (1995 ▶).
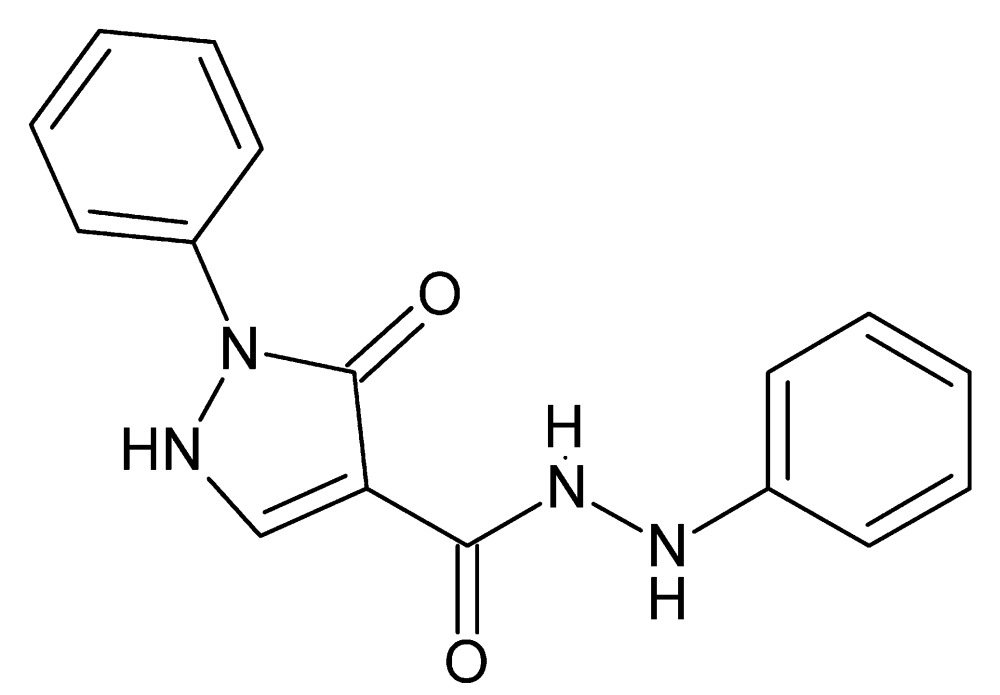


## Experimental   

### 

#### Crystal data  

C_16_H_14_N_4_O_2_
*M* *_r_* = 294.31
Monoclinic, 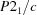

*a* = 8.4488 (12) Å
*b* = 11.5605 (17) Å
*c* = 14.642 (2) Å
β = 91.565 (2)°
*V* = 1429.6 (4) Å^3^
*Z* = 4
Mo *K*α radiation
μ = 0.09 mm^−1^
*T* = 150 K
0.26 × 0.20 × 0.07 mm

#### Data collection  

Bruker SMART APEX CCD diffractometer
Absorption correction: multi-scan (*SADABS*; Bruker, 2013 ▶) *T*_min_ = 0.85, *T*_max_ = 0.99
25571 measured reflections
3674 independent reflections
2895 reflections with *I* > 2σ(*I*)
*R*_int_ = 0.049

#### Refinement  

*R*[*F* ^2^ > 2σ(*F* ^2^)] = 0.043
*wR*(*F* ^2^) = 0.112
*S* = 1.03
3674 reflections
199 parameters
H-atom parameters constrained
Δρ_max_ = 0.25 e Å^−3^
Δρ_min_ = −0.21 e Å^−3^

### 

Data collection: *APEX2* (Bruker, 2013 ▶); cell refinement: *SAINT* (Bruker, 2013 ▶); data reduction: *SAINT*; program(s) used to solve structure: *SHELXTL* (Sheldrick, 2008 ▶); program(s) used to refine structure: *SHELXL2014* (Sheldrick, 2008 ▶); molecular graphics: *DIAMOND* (Brandenburg & Putz, 2012 ▶); software used to prepare material for publication: *SHELXTL*.

## Supplementary Material

Crystal structure: contains datablock(s) global, I. DOI: 10.1107/S1600536814012392/bt6982sup1.cif

Structure factors: contains datablock(s) I. DOI: 10.1107/S1600536814012392/bt6982Isup2.hkl

Supporting information file. DOI: 10.1107/S1600536814012392/bt6982Isup3.cml

CCDC reference: 1005600

Additional supporting information:  crystallographic information; 3D view; checkCIF report

## Figures and Tables

**Table 1:** Hydrogen-bond geometry (Å, °)

*D*—H⋯*A*	*D*—H	H⋯*A*	*D*⋯*A*	*D*—H⋯*A*
N1—H1*A*⋯O2^i^	0.91	2.06	2.9244 (14)	158
N2—H2*A*⋯O2	0.91	2.14	2.8597 (14)	136
N3—H3*A*⋯O1^ii^	0.91	1.75	2.6527 (15)	169
C12—H12⋯O2	0.95	2.28	2.9133 (18)	124
C16—H16⋯O1^ii^	0.95	2.51	3.2745 (18)	137

Symmetry codes: (i) 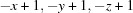; (ii) 
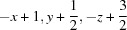
.

**Fig. 1.**
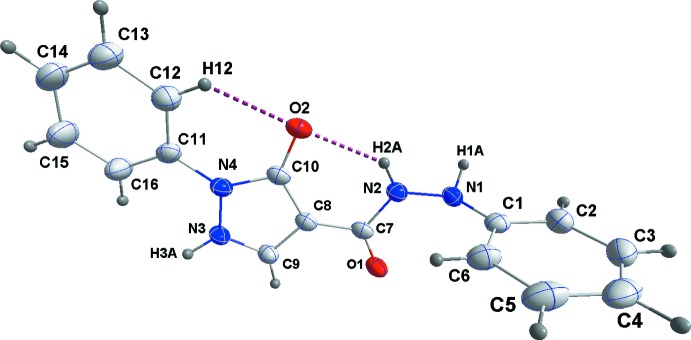
The title molecule showing intramolecular hydrogen bonds as dotted lines. Displacement ellipsoids are drawn at the 50% probability level.

**Fig. 2.**
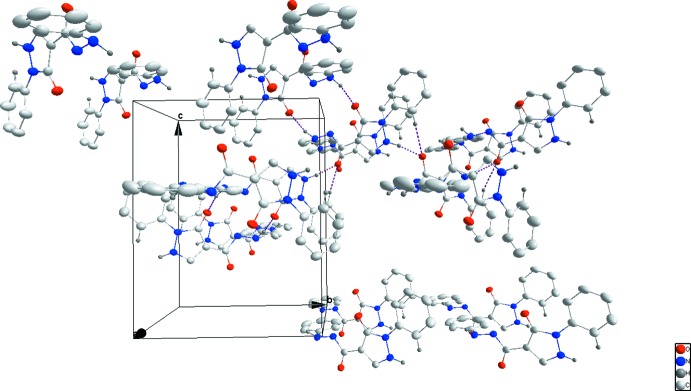
Packing viewed down the a axis with intermolecular hydrogen bonds shown as dotted lines.

**Fig. 3.**
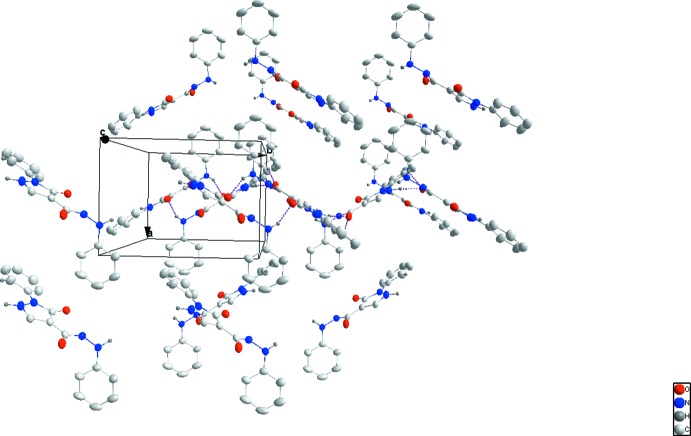
Packing viewed down the c axis with intermolecular hydrogen bonds shown as dotted lines.

## supplementary crystallographic information

### S1. Comment

Compounds containing a pyrazole core and related analogs have received signicant attention due to their chemical, medicinal, and pharmaceutical applications. Several reports showed the pyrazolone moiety to be one of most active pharmacophores and possesses anti-cancer (Guckian *et al*., 2010), anti-viral (Fan *et al.*, 2006), anti-tubercular (Castagnolo *et al.*, 2008), anti-hyperlipedaemic (Idrees *et al.*, 2009), anti-depressant, anti-convulsant (Abdel-Aziz *et al.*, 2009), anti-oxidant, anti-bacterial (Manojkumar *et al.*, 2009; Shete *et al*., 2011), anti-HIV (Sujatha *et al.*, 2009), anti-inflammatory, analgesic and anti-pyretic (El-Hawash *et al.*, 2006) activities. The pyrazolone-like edaravone has been developed as a drug for brain ischemia (Kawai *et al.*, 1997) and has also been reported to be effective for myocardial ischemia (Wu *et al.*, 2002). Additionally, pyrazolones have been reported to be the key starting materials for the synthesis of commercial aryl/heteroaropyrazolone dyes (Basaif *et al.*, 2007; Ho, 2005). Halogenated pyrazolones are also useful synthetic intermediates for synthesis of diazo-dyes (Kirschke *et al.*, 1984), fused (Chande *et al.*, 1993) and spiro-heterocyclic compounds (El-Saraf & El-Sayed 2003). In this context we report the synthesis and crystal structure of the title compound.

The phenyl ring C1–C6 is nearly perpendicular to the 5-membered ring (dihedral angle = 88.47 (5)°) while the latter makes a dihedral angle of 10.49 (8)° with the phenyl ring C11–C16. The rotational orientation of the latter phenyl ring as well as that of the N2—H2a unit are determined by the intramolecular C16—H16···O2 and N2—H2a···O2 hydrogen bonds (Table 1, Fig. 1) forming S(6) ring motifs (Bernstein *et al.*, 1995). The packing involves sheets of molecules parallel to the (100) plane formed by N3—H3a···O1 and pairwise N1—H1a···O2 intermolecular hydrogen bonds (Table 1, Figs. 2 and 3).

### S2. Experimental

A mixture of 10 mmol (2.31 g) of 4-[(dimethylamino)methylene]-1-phenylpyrazolidine-3,5-dione and 10 mmol (1.08 g) of phenyl hydrazine in 15 ml dioxane was refluxed for 6 h. After cooling, the resulting solid was collected by filtration and recrystallized from ethanol. Colourless crystals, 84%, m.p. = 483 K.

### S3. Refinement

H-atoms attached to carbon were placed in calculated positions (C—H = 0.95 Å) while those attached to nitrogen were placed in locations derived from a difference map and initially refined to check their identity following which their coordinates adjusted to give N—H = 0.91 Å. All were then included as riding contributions with isotropic displacement parameters 1.2 times those of the attached atoms.

### Crystal data

**Table:**

C_16_H_14_N_4_O_2_	*F*(000) = 616
*M**_r_* = 294.31	*D*_x_ = 1.367 Mg m^−^^3^
Monoclinic, *P*2_1_/*c*	Mo *K*α radiation, λ = 0.71073 Å
Hall symbol: -P 2ybc	Cell parameters from 9891 reflections
*a* = 8.4488 (12) Å	θ = 2.2–28.6°
*b* = 11.5605 (17) Å	µ = 0.09 mm^−^^1^
*c* = 14.642 (2) Å	*T* = 150 K
β = 91.565 (2)°	Plate, colourless
*V* = 1429.6 (4) Å^3^	0.26 × 0.20 × 0.07 mm
*Z* = 4	

### Data collection

**Table:**

Bruker SMART APEX CCD diffractometer	3674 independent reflections
Radiation source: fine-focus sealed tube	2895 reflections with *I* > 2σ(*I*)
Graphite monochromator	*R*_int_ = 0.049
Detector resolution: 8.3660 pixels mm^-1^	θ_max_ = 28.7°, θ_min_ = 2.2°
φ and ω scans	*h* = −11→11
Absorption correction: multi-scan (*SADABS*; Bruker, 2013)	*k* = −15→15
*T*_min_ = 0.85, *T*_max_ = 0.99	*l* = −19→19
25571 measured reflections	

### Refinement

**Table:**

Refinement on *F*^2^	Primary atom site location: structure-invariant direct methods
Least-squares matrix: full	Secondary atom site location: difference Fourier map
*R*[*F*^2^ > 2σ(*F*^2^)] = 0.043	Hydrogen site location: mixed
*wR*(*F*^2^) = 0.112	H-atom parameters constrained
*S* = 1.03	*w* = 1/[σ^2^(*F*_o_^2^) + (0.0439*P*)^2^ + 0.5339*P*] where *P* = (*F*_o_^2^ + 2*F*_c_^2^)/3
3674 reflections	(Δ/σ)_max_ < 0.001
199 parameters	Δρ_max_ = 0.25 e Å^−^^3^
0 restraints	Δρ_min_ = −0.21 e Å^−^^3^

### Special details

**Table:**

Geometry. Bond distances, angles *etc*. have been calculated using the rounded fractional coordinates. All su's are estimated from the variances of the (full) variance-covariance matrix. The cell e.s.d.'s are taken into account in the estimation of distances, angles and torsion angles
Refinement. Refinement on *F*^2^ for ALL reflections except those flagged by the user for potential systematic errors. Weighted *R*-factors *wR* and all goodnesses of fit *S* are based on *F*^2^, conventional *R*-factors *R* are based on *F*, with *F* set to zero for negative *F*^2^. The observed criterion of *F*^2^ > σ(*F*^2^) is used only for calculating -*R*-factor-obs *etc*. and is not relevant to the choice of reflections for refinement. *R*-factors based on *F*^2^ are statistically about twice as large as those based on *F*, and *R*-factors based on ALL data will be even larger.

### Fractional atomic coordinates and isotropic or equivalent isotropic displacement parameters (Å^2^)

**Table:**

	*x*	*y*	*z*	*U*_iso_*/*U*_eq_	
O1	0.63625 (12)	0.59121 (8)	0.76051 (6)	0.0326 (3)	
O2	0.48112 (11)	0.70165 (8)	0.48802 (6)	0.0318 (3)	
N1	0.72819 (12)	0.44304 (9)	0.62602 (7)	0.0271 (3)	
N2	0.64406 (12)	0.54510 (9)	0.60905 (7)	0.0271 (3)	
N3	0.38524 (14)	0.88161 (10)	0.66714 (7)	0.0304 (3)	
N4	0.38879 (13)	0.85733 (9)	0.57481 (7)	0.0280 (3)	
C1	0.89573 (15)	0.45011 (12)	0.62204 (8)	0.0284 (3)	
C2	0.98148 (18)	0.34748 (14)	0.62651 (10)	0.0394 (4)	
C3	1.14618 (19)	0.35060 (17)	0.62340 (11)	0.0501 (5)	
C4	1.22455 (18)	0.45435 (18)	0.61602 (10)	0.0506 (6)	
C5	1.13945 (18)	0.55579 (17)	0.61308 (10)	0.0468 (5)	
C6	0.97471 (16)	0.55477 (13)	0.61649 (9)	0.0357 (4)	
C7	0.60259 (15)	0.61338 (10)	0.67917 (8)	0.0259 (3)	
C8	0.51543 (15)	0.71675 (11)	0.65311 (8)	0.0263 (3)	
C9	0.46389 (16)	0.79948 (11)	0.71259 (8)	0.0285 (4)	
C10	0.46608 (15)	0.75198 (11)	0.56291 (8)	0.0264 (3)	
C11	0.31419 (16)	0.93191 (11)	0.50934 (8)	0.0294 (3)	
C12	0.3405 (2)	0.91486 (13)	0.41745 (9)	0.0401 (5)	
C13	0.2658 (2)	0.98677 (15)	0.35349 (10)	0.0470 (5)	
C14	0.1706 (2)	1.07633 (15)	0.37982 (11)	0.0456 (5)	
C15	0.1489 (2)	1.09488 (16)	0.47180 (11)	0.0507 (6)	
C16	0.21914 (19)	1.02231 (14)	0.53697 (10)	0.0429 (5)	
H1A	0.68570	0.38480	0.59150	0.0320*	
H2	0.92800	0.27560	0.63170	0.0470*	
H2A	0.62470	0.57050	0.55100	0.0320*	
H3	1.20480	0.28050	0.62640	0.0600*	
H3A	0.36760	0.95490	0.68710	0.0360*	
H4	1.33670	0.45590	0.61300	0.0610*	
H5	1.19360	0.62750	0.60870	0.0560*	
H6	0.91710	0.62530	0.61500	0.0430*	
H9	0.48190	0.79820	0.77690	0.0340*	
H12	0.40880	0.85480	0.39830	0.0480*	
H13	0.28090	0.97360	0.29030	0.0560*	
H14	0.12040	1.12490	0.33540	0.0550*	
H15	0.08530	1.15780	0.49080	0.0610*	
H16	0.20200	1.03470	0.60010	0.0520*	

### Atomic displacement parameters (Å^2^)

**Table:**

	*U*^11^	*U*^22^	*U*^33^	*U*^12^	*U*^13^	*U*^23^
O1	0.0487 (6)	0.0260 (5)	0.0230 (4)	−0.0009 (4)	0.0018 (4)	−0.0005 (3)
O2	0.0371 (5)	0.0345 (5)	0.0239 (4)	−0.0022 (4)	0.0012 (4)	−0.0072 (4)
N1	0.0270 (5)	0.0257 (5)	0.0286 (5)	−0.0012 (4)	0.0029 (4)	−0.0043 (4)
N2	0.0299 (5)	0.0286 (5)	0.0229 (5)	0.0003 (4)	0.0018 (4)	−0.0019 (4)
N3	0.0432 (6)	0.0268 (5)	0.0212 (5)	0.0004 (5)	0.0013 (4)	−0.0033 (4)
N4	0.0355 (6)	0.0275 (5)	0.0209 (5)	−0.0029 (4)	0.0012 (4)	−0.0026 (4)
C1	0.0268 (6)	0.0398 (7)	0.0186 (5)	−0.0005 (5)	0.0015 (4)	−0.0041 (5)
C2	0.0369 (7)	0.0458 (8)	0.0354 (7)	0.0058 (6)	−0.0009 (6)	−0.0100 (6)
C3	0.0378 (8)	0.0735 (12)	0.0386 (8)	0.0187 (8)	−0.0043 (6)	−0.0178 (8)
C4	0.0271 (7)	0.0945 (14)	0.0300 (7)	−0.0009 (8)	−0.0005 (6)	−0.0155 (8)
C5	0.0343 (8)	0.0735 (12)	0.0326 (7)	−0.0175 (8)	−0.0005 (6)	−0.0012 (7)
C6	0.0314 (7)	0.0462 (8)	0.0294 (6)	−0.0083 (6)	−0.0002 (5)	0.0010 (6)
C7	0.0286 (6)	0.0248 (6)	0.0243 (6)	−0.0078 (5)	0.0027 (5)	−0.0024 (5)
C8	0.0301 (6)	0.0249 (6)	0.0239 (6)	−0.0053 (5)	0.0027 (5)	−0.0020 (4)
C9	0.0371 (7)	0.0258 (6)	0.0227 (6)	−0.0027 (5)	0.0013 (5)	−0.0007 (5)
C10	0.0278 (6)	0.0269 (6)	0.0246 (6)	−0.0065 (5)	0.0028 (5)	−0.0034 (5)
C11	0.0323 (6)	0.0303 (6)	0.0254 (6)	−0.0079 (5)	−0.0029 (5)	0.0012 (5)
C12	0.0551 (9)	0.0380 (8)	0.0274 (7)	−0.0010 (7)	0.0031 (6)	0.0003 (6)
C13	0.0633 (10)	0.0502 (9)	0.0272 (7)	−0.0051 (8)	−0.0014 (7)	0.0052 (6)
C14	0.0510 (9)	0.0471 (9)	0.0378 (8)	−0.0043 (7)	−0.0130 (7)	0.0098 (7)
C15	0.0572 (10)	0.0515 (10)	0.0427 (9)	0.0147 (8)	−0.0115 (7)	−0.0006 (7)
C16	0.0503 (9)	0.0473 (9)	0.0307 (7)	0.0106 (7)	−0.0064 (6)	−0.0034 (6)

### Geometric parameters (Å, º)

**Table:**

O1—C7	1.2436 (15)	C8—C9	1.3724 (18)
O2—C10	1.2509 (15)	C8—C10	1.4332 (17)
N1—N2	1.3962 (15)	C11—C16	1.385 (2)
N1—C1	1.4207 (16)	C11—C12	1.3838 (18)
N2—C7	1.3489 (16)	C12—C13	1.391 (2)
N3—N4	1.3819 (15)	C13—C14	1.373 (2)
N3—C9	1.3273 (17)	C14—C15	1.381 (2)
N4—C10	1.3951 (17)	C15—C16	1.391 (2)
N4—C11	1.4236 (16)	C2—H2	0.9500
N1—H1A	0.9100	C3—H3	0.9500
N2—H2A	0.9100	C4—H4	0.9500
N3—H3A	0.9100	C5—H5	0.9500
C1—C6	1.385 (2)	C6—H6	0.9500
C1—C2	1.391 (2)	C9—H9	0.9500
C2—C3	1.394 (2)	C12—H12	0.9500
C3—C4	1.376 (3)	C13—H13	0.9500
C4—C5	1.376 (3)	C14—H14	0.9500
C5—C6	1.394 (2)	C15—H15	0.9500
C7—C8	1.4493 (17)	C16—H16	0.9500
			
N2—N1—C1	116.56 (10)	N4—C11—C12	119.25 (12)
N1—N2—C7	120.04 (10)	N4—C11—C16	120.65 (11)
N4—N3—C9	108.74 (10)	C12—C11—C16	120.09 (13)
N3—N4—C10	108.80 (10)	C11—C12—C13	119.18 (14)
N3—N4—C11	120.98 (10)	C12—C13—C14	121.35 (14)
C10—N4—C11	130.15 (10)	C13—C14—C15	119.04 (15)
N2—N1—H1A	110.00	C14—C15—C16	120.65 (16)
C1—N1—H1A	113.00	C11—C16—C15	119.64 (14)
C7—N2—H2A	119.00	C1—C2—H2	120.00
N1—N2—H2A	121.00	C3—C2—H2	120.00
N4—N3—H3A	121.00	C2—C3—H3	120.00
C9—N3—H3A	126.00	C4—C3—H3	120.00
C2—C1—C6	119.79 (12)	C3—C4—H4	120.00
N1—C1—C2	117.86 (12)	C5—C4—H4	120.00
N1—C1—C6	122.33 (12)	C4—C5—H5	120.00
C1—C2—C3	119.71 (15)	C6—C5—H5	120.00
C2—C3—C4	120.54 (17)	C1—C6—H6	120.00
C3—C4—C5	119.57 (15)	C5—C6—H6	120.00
C4—C5—C6	120.87 (16)	N3—C9—H9	125.00
C1—C6—C5	119.50 (14)	C8—C9—H9	125.00
N2—C7—C8	115.01 (10)	C11—C12—H12	120.00
O1—C7—N2	123.43 (11)	C13—C12—H12	120.00
O1—C7—C8	121.56 (11)	C12—C13—H13	119.00
C7—C8—C10	127.58 (11)	C14—C13—H13	119.00
C9—C8—C10	107.30 (11)	C13—C14—H14	120.00
C7—C8—C9	125.12 (11)	C15—C14—H14	120.00
N3—C9—C8	110.09 (11)	C14—C15—H15	120.00
N4—C10—C8	104.97 (10)	C16—C15—H15	120.00
O2—C10—C8	129.92 (12)	C11—C16—H16	120.00
O2—C10—N4	125.08 (11)	C15—C16—H16	120.00
			
C1—N1—N2—C7	−93.97 (13)	C2—C3—C4—C5	1.0 (2)
N2—N1—C1—C2	−171.62 (11)	C3—C4—C5—C6	−0.7 (2)
N2—N1—C1—C6	10.19 (17)	C4—C5—C6—C1	−0.6 (2)
N1—N2—C7—O1	0.51 (18)	O1—C7—C8—C9	0.9 (2)
N1—N2—C7—C8	−179.89 (10)	O1—C7—C8—C10	−178.68 (13)
C9—N3—N4—C10	3.27 (14)	N2—C7—C8—C9	−178.74 (12)
C9—N3—N4—C11	−179.56 (11)	N2—C7—C8—C10	1.71 (19)
N4—N3—C9—C8	−2.57 (15)	C7—C8—C9—N3	−178.73 (12)
N3—N4—C10—O2	175.50 (12)	C10—C8—C9—N3	0.90 (15)
N3—N4—C10—C8	−2.61 (13)	C7—C8—C10—O2	2.7 (2)
C11—N4—C10—O2	−1.3 (2)	C7—C8—C10—N4	−179.30 (12)
C11—N4—C10—C8	−179.44 (12)	C9—C8—C10—O2	−176.90 (13)
N3—N4—C11—C12	170.15 (13)	C9—C8—C10—N4	1.08 (14)
N3—N4—C11—C16	−8.37 (19)	N4—C11—C12—C13	179.11 (14)
C10—N4—C11—C12	−13.3 (2)	C16—C11—C12—C13	−2.4 (2)
C10—N4—C11—C16	168.14 (13)	N4—C11—C16—C15	179.27 (14)
N1—C1—C2—C3	−179.61 (13)	C12—C11—C16—C15	0.8 (2)
C6—C1—C2—C3	−1.4 (2)	C11—C12—C13—C14	2.0 (2)
N1—C1—C6—C5	179.80 (12)	C12—C13—C14—C15	−0.1 (3)
C2—C1—C6—C5	1.64 (19)	C13—C14—C15—C16	−1.6 (3)
C1—C2—C3—C4	0.1 (2)	C14—C15—C16—C11	1.2 (3)

### Hydrogen-bond geometry (Å, º)

**Table:**

*D*—H···*A*	*D*—H	H···*A*	*D*···*A*	*D*—H···*A*
N1—H1*A*···O2^i^	0.91	2.06	2.9244 (14)	158
N2—H2*A*···O2	0.91	2.14	2.8597 (14)	136
N3—H3*A*···O1^ii^	0.91	1.75	2.6527 (15)	169
C12—H12···O2	0.95	2.28	2.9133 (18)	124
C16—H16···O1^ii^	0.95	2.51	3.2745 (18)	137

Symmetry codes: (i) −*x*+1, −*y*+1, −*z*+1; (ii) −*x*+1, *y*+1/2, −*z*+3/2.
